# Effect of environmental contamination on female and male gametes – A lesson from bovines

**DOI:** 10.1590/1984-3143-AR2020-0041

**Published:** 2020-08-05

**Authors:** Zvi Roth, Alisa Komsky-Elbaz, Dorit Kalo

**Affiliations:** a Department of Animal Sciences, The Robert H. Smith Faculty of Agriculture, Food and Environment, The Hebrew University of Jerusalem, Rehovot 76100, Israel

**Keywords:** environmental contaminant, bovine, oocyte, spermatozoa, embryonic development

## Abstract

Endocrine-disrupting compounds (EDCs) and foodborne contaminants are environmental pollutants that are considered reproductive toxicants due to their deleterious effects on female and male gametes. Among the EDCs, the phthalate plasticizers are of growing concern. In-vivo and in-vitro models indicate that the oocyte is highly sensitive to phthalates. This review summarizes the effects of di(2-ethylhexyl) phthalate and its major metabolite mono(2-ethyhexyl) phthalate (MEHP) on the oocyte. MEHP reduces the proportion of oocytes that fertilize, cleave and develop to the blastocyst stage. This is associated with negative effects on meiotic progression, and disruption of cortical granules, endoplasmic reticulum and mitochondrial reorganization. MEHP alters mitochondrial membrane polarity, increases reactive oxygen species levels and induces alterations in genes associated with oxidative phosphorylation. A carryover effect from the oocyte to the blastocyst is manifested by alterations in the transcriptomic profile of blastocysts developed from MEHP-treated oocytes. Among foodborne contaminants, the pesticide atrazine (ATZ) and the mycotoxin aflatoxin B1 (AFB1) are of high concern. The potential hazards associated with exposure of spermatozoa to these contaminants and their carryover effect to the blastocyst are described. AFB1 and ATZ reduce spermatozoa's viability, as reflected by a high proportion of cells with damaged plasma membrane; induce acrosome reaction, expressed as damage to the acrosomal membrane; and interfere with mitochondrial function, characterized by hyperpolarization of the membrane. ATZ and AFB1-treated spermatozoa show a high proportion of cells with fragmented DNA. Exposure of spermatozoa to AFB1 and ATZ reduces fertilization and cleavage rates, but not that of blastocyst formation. However, fertilization with AFB1- or ATZ-treated spermatozoa impairs transcript expression in the formed blastocysts, implying a carryover effect. Taken together, the review indicates the risk of exposing farm animals to environmental contaminants, and their deleterious effects on female and male gametes and the developing embryo.

## Introduction

Female and male reproductive health can be affected by various environmental stressors. While much attention has been paid to the increase in ambient temperature, i.e., global warming, less attention has been given to environmental contamination and its consequences on animal fertility. Reproductive health can be affected by a variety of manmade environmental contaminants (Diamanti-Kandarakis et al., 2009) or foodborne toxins (Jin et al., 2015; Pflieger-Bruss et al., 2004), some of which are defined as endocrine-disrupting compounds (EDCs).

EDCs are exogenous chemicals that can interfere with natural hormone activities, such as synthesis, secretion, transport, metabolism, and binding action, thereby impairing homeostasis, reproduction, and developmental processes. EDCs act by binding to nuclear receptors [e.g., aryl-hydrocarbon (dioxin) receptor, peroxisome proliferator-activated receptor family]. EDCs can also act via other mechanisms that are associated with endocrine and reproductive systems by binding to non-nuclear steroid hormone receptors (e.g., membrane estrogen and androgen receptors), non-steroid receptors (e.g., serotonin, dopamine and norepinephrine receptor) (Diamanti-Kandarakis et al., 2009).

Throughout the female’s lifetime, follicular growth is characterized by recruitment of a cohort of primordial follicles into the pool of growing follicles from which a preovulatory follicle develops. In bovine, this course of follicular development is lengthy, taking approximately 180 days (Lussier et al., 1987). Therefore, exposure to environmental contaminants during early stages of follicular development might further affect the function of the follicle and its enclosed oocytes. EDCs act on the hypothalamic–pituitary–ovarian axis, and their effect on the female reproductive tract is therefore multifactorial in nature. The deleterious effect is expressed as ovarian failure, impaired follicular development, accelerated atresia and depletion of antral growing follicles, ovulation failure and impaired steroidogenesis. These, in turn, might lead to reduced fertility (Bhattacharya and Keating, 2012; Craig et al., 2011; Zama et al., 2016).

Similarly, spermatogenesis is a highly intricate and coordinated process that produces thousands of spermatozoa daily in the seminiferous tubules of the testes, which then pass through the epididymal caput (head) and corpus (body) and are stored in the cauda (tail) until ejaculation (Reyes-Moreno et al., 2002; Cornwall, 2014). In the bull, this process takes about 60 days (Staub and Johnson, 2018). During their passage through the epididymis compartments, the spermatozoa undergo extensive changes, which are completed in the female reproductive tract during the capacitation process. Given that spermatogenesis is also a lengthy process, spermatozoa are potentially exposed to a variety of environmental stressors at their different developmental stages. Indeed, epidemiological studies suggest an association between exposure to pesticides and semen quality (Swan et al., 2003). In that respect, the global decline in male fertility is mainly attributed to the exposure of spermatozoa to environmental EDCs, pharmaceutical products, pesticides and mycotoxins of food origin (Eze et al., 2018; Mann et al., 2020; Tavares et al., 2016).

The aim of the current review is to describe the risk associated with exposing farm animals to environmental contaminants. The review focuses on the effects of representative EDCs on female and male gametes, in particular the effects of the plasticizers phthalates on the oocyte, and of foodborne contaminants on the spermatozoa. A carryover effect from the gametes to the developed embryo is also discussed.

## Endocrine-disrupting compounds

The list of EDCs consists of more than 80,000 heterogeneous chemicals that are used for numerous applications. Some of these are the polychlorinated biphenyls, polybrominated biphenyls, dioxins, plastics (bisphenol A;BPA), pesticides [methoxychlor, chlorpyrifos, atrazine (ATZ), dichlorodiphenyltrichloroethane (DDT)], fungicides (vinclozolin), pharmaceutical agents (diethylstilbestrol) and plasticizers (phthalates) (Diamanti-Kandarakis et al., 2009). The current section focuses on phthalates, a group of chemical reproductive toxicants, and discusses the mechanism by which phthalates impair oocyte competence and further, the formed embryo.

## Phthalates

Phthalate esters have been widely used in manufacturing for about a century (Koch and Calafat, 2009). The annual production of phthalate is estimated at over 6 million tons worldwide, making these chemicals ubiquitous (Net et al., 2015a, b). More than 25 types of phthalates are used for commercial applications such as toys, vinyl flooring, wall covering, detergents, lubricating oils, food packaging, pharmaceuticals, blood bags and personal care products (Koch and Calafat, 2009). The top 10 are dimethyl phthalate, diethyl phthalate, dibutyl phthalate, diisobutyl phthalate, benzylbutyl phthalate, dicyclohexyl phthalate, di(2-ethylhexyl) phthalate (DEHP), di-n-octyl phthalate, di-isononyl phthalate, and di-isodecyl phthalate. With respect to animal exposure, in particular dairy cows, phthalates can be found in milking equipment such as milking pipes, liners, teat dip cups, food packing and silage wrap (Pure Strategies, 2018).

Among phthalates, DEHP is predominant, providing softness and flexibility to polyvinyl chloride products (ATSDR, 2002). DEHP does not bind covalently to the polyvinyl chloride polymer; it therefore leaches into the environment and is frequently found in the atmosphere, soil, sediment, and water sources (Bergé et al., 2013; Hongjun et al., 2013; Martine et al., 2013; Net et al., 2015b). It is estimated that humans are exposed to DEHP at around 1.7–52.1 µg/kg body weight (BW) per day (McKee et al., 2004; Petersen and Breindahl, 2000). Infants are suggested to be at higher risk under certain circumstances (Kavlock et al., 2006). In the body, DEHP is metabolized into several metabolites, including mono(2-ethyl-5-hydroxyhexyl) phthalate (5OH-MEHP), mono(2-ethyl-5-carboxypentyl) phthalate (5cx-MEPP), mono(2-ethyl-5-oxohexyl) phthalate (5oxo-MEHP), mono[2-(carboxymethyl)hexyl] phthalate (2cx-MMHP), and mono(2-ethyhexyl) phthalate (MEHP) (Koch et al., 2005). According to the US Food and Drug Administration, the metabolites have more adverse and toxic effects than DEHP.

While the effects of phthalates on human health have been extensively studied (Benjamin et al., 2017; Hauser and Calafat, 2005), the magnitude and risk of exposure of domestic animals to phthalates is less known, most likely due to less awareness or concern. Nevertheless, recent studies have reported a deleterious effect of phthalates on animal health. In vivo studies in farm animals demonstrated the clearance pattern of DEHP. Oral administration to young male pigs of 1000 mg/kg BW DEHP resulted in a rapid increase in MEHP level (14 ng/L) in the blood with a half-life of ~6 h (Ljungvall et al., 2004). Oral administration of DEHP to cows resulted in low levels of the metabolites 5OH-MEHP, MEHP, 5oxo-MEHP, 2cx-MMHP, and 5cx-MEPP were found in the milk, urine, and plasma samples of DEHP-treated cows, 20 days after DEHP administration (Kalo et al., 2015). A level of ~20 nM MEHP was detected in the follicular fluid aspirated from the DEHP-treated cows (Kalo et al., 2015). Similarly, a level of ~10 nM DEHP was recorded in equine follicular fluid (Marzano et al., 2019). Non-DEHP-originated metabolites such as monoethyl phthalate and monobutyl phthalate were also detected in the follicular fluid, presumably of environmental origin (Kalo et al., 2015). In addition, DEHP has been reported in bovine milk (Jarošová, 2006; Krejčíková and Jarošová, 2013) and fat tissue (Jarošová, 2006) and in ewe (Rhind et al., 2005) and swine tissues (Jarošová, 2006; Ljungvall et al., 2004).

## Effect of phthalates on female reproduction

There is accumulating evidence associating the presence of phthalates in the environment with impaired reproduction and reduced female fertility. In women undergoing in-vitro fertilization treatments, the presence of DEHP and its metabolites in the urine has been associated with a low number of retrieved, matured and fertilized oocytes and a reduced number of high-quality embryos (Machtinger et al., 2018). Exposure of women to MEHP close to conception was associated with early pregnancy loss (Toft et al., 2011). High levels of MEHP and 5oxo-MEHP in the urine have been associated with long gestation and interruption in parturition (Adibi et al., 2009). Tranfo et al. (2012) found a correlation between the level of DEHP metabolites in the urine and infertility, in both men and women. In mice, DEHP and MEHP deleteriously affected the ovarian pool of follicles, expressed as a reduction in the number of primordial and antral follicles (Hannon et al., 2016; Li et al., 2016; Moyer and Hixon, 2012; Niermann et al., 2015; Zhang et al., 2013). Chronic exposure of female mice, at implantation, to a high level of phthalate (250 mg/kg BW) was associated with decreased pregnancy rates and increased miscarriage rates (Aldyreva et al., 1975). Exposure of female rats to high doses of DEHP (up to 2 g/kg BW) altered estradiol concentration, inhibited follicular growth, disrupted the estrous cycle, and impaired ovulation (Davis et al., 1994; Gupta et al., 2010; Lovekamp-Swan and Davis, 2003). With respect to DEHP metabolites, studies in mice have reported that MEHP inhibits follicular growth and reduces estradiol production in antral follicles (Gupta et al., 2010; Wang et al., 2012). In vivo exposure of cows to 100 mg/kg DEHP per day for 3 days impairs the pattern of follicular development, decreases estradiol concentration and increase follicular pathologies (Kalo et al., 2015).

Taken together, it is becoming clear that both DEHP and MEHP are reproductive toxicants (Balabanič et al., 2011; Heudorf et al., 2007; Johnson et al., 2012; Kay et al., 2013).

## Effects of DEHP and MEHP on the oocyte

Gathering evidence indicates that the oocyte is highly sensitive to phthalates. Oral administration of mice with 50–200 µL of 2.56 µM DEHP for 12 days reduced the number of mature oocytes, and decreased fertilization rate and embryonic development (Eimani et al., 2005). Exposing the ovaries of newborn mice, in vitro, to DEHP (10 or 100 µM) induced apoptosis, as it increased the mRNA expression of *Bax,* a proapoptotic factor, and the proportion of TUNEL-positive oocytes (Zhang et al., 2014). Oral administration of DEHP (50, 100 or 200 μL of 2.56 μM) to mice dose-dependently reduced the number of oocytes progressing to metaphase II. This was accompanied by a decrease in oocyte developmental competence, an increase in early apoptosis, and a decrease in the expression levels of the genes *Ccna1, Asah1* and *Pou5f1* (Absalan et al., 2017). Oral administration of 2000 μg/kg BW DEHP per day to mice resulted in a lower number of retrieved oocytes, a higher number of unfertilized oocytes, a higher number of fragmented zygotes, and decreased embryonic development (Parra-Forero et al., 2019).

While much data have been reported for rodents, data from farm animals are limited. Acute exposure of equine oocytes to DEHP (12 and 1200 µM) induced apoptosis and increased reactive oxygen species (ROS) levels in the cumulus cells, but did not affect nuclear maturation (Ambruosi et al., 2011). In-vitro culture of equine oocytes with 0.12 µM DEHP inhibited their nuclear maturation, and increased apoptosis and ROS levels in the surrounding cumulus cells (Ambruosi et al., 2011). On the other hand, exposure of porcine cumulus oocyte complexes (COCs) to extremely high doses of DEHP (250 µM to 5 mM) did not affect the proportion of oocytes with extruded polar body (Wang et al., 2016). Exposure of bovine COCs to 50 µM DEHP during oocyte maturation did not affect their cleavage rate into 2- to 4-cell-stage embryos, but decreased blastocyst-formation rate (Grossman et al., 2012).

**Figure 1:**
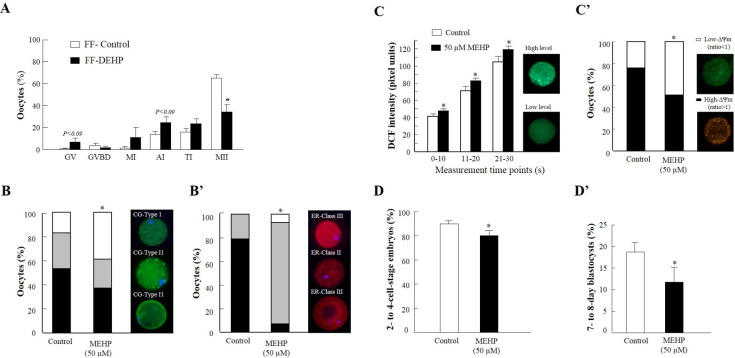
Effect of the EDC MEHP on bovine oocytes. (A) Nuclear maturation of oocytes cultured in follicular fluid (FF) aspirated from control (FF-control) and DEHP-treated (FF-DEHP) cows. Presented is the distribution of oocytes into meiotic stages: germinal vesicle (GV), GV breakdown (GVBD), metaphase I (MI), anaphase I (AI), telophase I (TI), and metaphase II (MII) (adapted with permission from Kalo et al., 2015). (B,B') Cytoplasmic maturation is affected by MEHP. (B) Distribution of mature oocytes among three types of cortical granule (CG) patterns (fluorescein-conjugated peanut agglutinin [FITC–PNA] dye): CG-Type I (immature), CG-Type II (partially mature) and CG-Type III (fully mature). (B') Distribution of mature oocytes among three classes of endoplasmic reticulum (ER) pattern (red dye): ER-Class III (degenerative), ER-Class II (immature) and ER-Class I (fully mature). (C,C') Mitochondrial function is affected by MEHP. (C) ROS level expressed by dichlorofluorescein (DCF) fluorescence intensity, measured from 0–10, 11–20 and 21–30 s in mature oocytes, and representative images of live oocyte stained with dichlorodihydrofluorescein diacetate (H_2_DCFDA) expressing low (bottom) or high (top) DCF fluorescence intensity. (C') Proportion of oocytes with high- and low-polarized mitochondrial membrane (∆Ψm) along with representative images of live oocytes stained with MitoCapture mitochondrial apoptosis kit expressing high (red) and low (green) ∆Ψm (adapted with permission from Kalo and Roth, 2015). (D,D') Developmental competence of oocytes in-vitro matured without or with 50 µM MEHP, further fertilized and cultured for 8 days. (D) Cleavage rate into 2- to 4-cell-stage embryos. (D') Blastocyst-formation rate (adopted with permission from Grossman et al., 2012).

In-vitro exposure of bovine oocytes to MEHP (50 µM) impaired nuclear maturation, reflected by a decreased proportion of oocytes that resumed meiosis and progressed to the metaphase II stage (Grossman et al., 2012; Kalo and Roth, 2015). In-vitro culturing of bovine oocytes in follicular fluid containing ~20 nM MEHP resulted in a low proportion of oocytes that resumed meiosis. In particular, a higher proportion of MEHP-treated oocytes were blocked in the telophase I stage (Figure 1A; Kalo et al., 2015). In support of this, exposure of COCs or denuded bovine oocytes to 5–100 µM MEHP increased the proportion of oocytes expressing a germinal vesicle stage nucleus by the end of culture, i.e., they did not resume meiosis (Anas et al., 2003). Similar findings were reported for bovine COCs cultured with a high concentration of MEHP (75 µM) (Beker van Woudenberg et al., 2012) and for porcine COCs cultured with 50–100 μM MEHP (Zhang et al., 2018).

One of the suggested mechanisms by which phthalates impair oocyte nuclear maturation is alterations in the cAMP-signaling pathway. An accurate cAMP level is required for chromatin transition, and synchronization between nuclear and cytoplasmic maturation (Gilchrist et al., 2011; Luciano et al., 2011). Microarray analysis of MEHP-treated bovine oocytes revealed alterations in seven transcripts associated with the cAMP-signaling pathway (Kalo et al., 2019). In rats, MEHP inhibited the accumulation of cAMP in Sertoli cells (Heindel and Chapin, 1989). In addition, exposure of rat granulosa cells to 100 µM MEHP decreased progesterone production and follicle-stimulating hormone-induced cAMP accumulation (Treinen et al., 1990). MEHP-induced impairment of the cell cycle has also been suggested as a potential mechanism, because exposure of bovine oocytes to 50 µM MEHP altered the expression level of *CCNA2* (Grossman et al., 2012). In mice, oral administration of 100 µL DEHP or MEHP (2.56 µM) resulted in reduced expression of the pluripotency (*Pou5f1*), cell cycle (*Ccna1*) and apoptotic (*Asah1)* related genes (Absalan et al., 2017).

Recent studies have provided evidence that phthalates induce alterations in cytoplasmic maturation in association with reduced oocyte competence. The maturation process includes multiple activities that are highly important to the formation of a mature and fertilizable oocyte. These include organelle redistribution (mitochondria, endoplasmic reticulum, cortical granules and lipid droplets), cytoskeletal rearrangement, and cytoplasmic polyadenylation (Eppig, 1996; Ferreira et al., 2009). Culturing of bovine COCs with 50 µM MEHP altered oocyte cytoplasmic maturation, reflected by alterations in the reorganization of the mitochondria, cortical granules and endoplasmic reticulum (Figure 1B,B'; Kalo and Roth, 2015). Given the pivotal function of the mitochondria in early stages of embryonic development (Acton et al., 2004; Bavister and Squirrell, 2000; Thouas et al., 2004), MEHP-induced impairment in oocyte mitochondrial function is suggested to be one of the main mechanisms underlying reduced developmental competence (Roth, 2018). Alterations in mitochondrial distribution were associated with increased ROS levels and decreased mitochondrial membrane potential (ΔΨm) (Figure 1C,C': Kalo and Roth, 2015). In mice, oral treatment with DEHP (40 μg/kg BW) increased ROS levels in the oocytes, along with disruption of mitochondrial function and reduction in ATP levels (Lu et al., 2019). In equine, an increase in ROS and ATP levels was recorded in oocytes that were exposed to 12 µM DEHP (Ambruosi et al., 2011). Zhang et al. (2018) reported that in-vitro culturing of porcine COCs with 75 µM MEHP results in increased ROS production.

Changes in mitochondrial function along with the increased levels of ROS might further lead to apoptosis. Culturing of bovine COCs with 50 µM MEHP increased the proportion of apoptotic oocytes (TUNEL+) (Kalo and Roth, 2015) and was associated with alteration in the expression of *ASAH1* gene encodes the acid ceramidase enzyme, an antiapoptotic factor (Grossman et al., 2012). In-depth transcript screening and proteomic analysis strongly support the oocyte transcripts' sensitivity to MEHP, since alterations in several genes associated with mitochondria and the apoptotic cascade have been reported (Kalo et al., 2019). In support of this, MEHP induced alterations in the expression of genes associated with mitochondrial function in bovine COCs (cytochrome bc1 complex; *CYC1*, Mitochondrially Encoded Cytochrome C Oxidase I ;*MT-CO1* and ATP synthase subunit beta; *ATP5B*), mouse fetal oocytes (superoxide dismutase 1 ;*Sod1* and NADH dehydrogenase 1; *Nd1*) and porcine COCs (*SOD1* and Catalase; *CAT*) (Kalo and Roth, 2015; Bonilla and del Mazo, 2010; Zhang et al., 2018).

## Carryover effect of DEHP and MEHP from the oocyte to the embryo

The early developing embryo, as well as the growing fetus, can also be exposed to phthalates during their growth in the uterus. In humans, DEHP and MEHP have been detected in maternal serum and cord blood, peritoneal fluid, meconium and amniotic fluid (Arbuckle et al., 2016; Huang et al., 2014; Latini et al., 2003; Silva et al., 2004). Oral administration of DEHP (10 mg/kg BW) to female mice on day 7 or 8 of gestation led to a high incidence of death or malformed fetuses, whereas exposure before or after these crucial days resulted in fewer defective fetuses (Yagi et al., 1980). Exposure of mice on day 8 of gestation to 1 mL/kg BW MEHP resulted in skeletal abnormalities and fetal deaths (Tomita et al., 1982; Yagi et al., 1980). In-vitro culture of 8-day-old mouse embryos with DEHP and MEHP (100–1000 µg/mL) impaired their development and increased the proportion of malformed embryos (Sant et al., 2016).

Moreover, carryover effects from the oocyte to the developed embryo have been documented. Culturing of bovine COCs with either 50 µM DEHP or MEHP resulted in reduced oocyte developmental competence, expressed as a lower cleavage rate and reduced blastocyst-formation rate (Figure 1D, D'; Grossman et al., 2012). In-vitro exposure of bovine oocytes to relatively low levels of MEHP (20–1000 nM) decreased their developmental competence, reflected by a low proportion of oocytes that fertilized, cleaved and developed to the blastocyst stage (Kalo and Roth, 2017). In-vitro maturation of oocytes in follicular fluid aspirated from DEHP-treated cows (i.e., containing 20 nM MEHP) reduced cleavage and blastocyst-formation rates (Kalo et al., 2015). Blastocysts developed from MEHP-treated oocytes showed different expression patterns of genes involved in intracellular mechanisms such as pluripotency, apoptosis, and placental development (Kalo et al., 2019). Culturing of bovine oocytes with 50 µM MEHP altered the levels of *POU5F1* in 2-cell-stage embryos (Grossman et al., 2012). MEHP-induced impairment in *POU5F1* and *SOX2* transcript abundance which associated with pluripotency, was found in blastocysts developed from oocytes exposed to relatively low MEHP concentrations (Kalo and Roth, 2017), suggesting that these embryos are of inferior quality. In-vitro maturation of bovine oocytes with 20 nM MEHP induced a carryover effect on the transcriptomic profile of the developing embryo, expressed by the altered expression of 260 genes (Kalo et al., 2019). Direct exposure of mouse embryos to 100–1000 μg/mL MEHP (corresponding to 0.4–3.6 mM) impaired the expression of metabolic genes, in particular those related to the electron transport chain (Sant et al., 2016). In agreement with this, a carryover effect was also recorded in female mice treated with DEHP or MEHP, manifested in a reduction in the proportion of developing blastocysts (Absalan et al., 2017).

## Summary

This section provides broad information on the direct and indirect effects of phthalates on ovarian function, oocyte maturation and embryonic developmental competence. Note that when the oocyte was exposed to MEHP, deleterious effects were further recorded in the developed embryos. In addition, phthalate-induced damage involves multiple mechanisms which might also be relevant to other EDCs.

## Foodborne contaminants

A wide range of chemicals have been identified as foodborne contaminants that can be potentially found in food and water and might be consumed by humans and/or animals. These include pesticides and herbicides (ATZ, methoxychlor, chlorpyrifos, DDT) (Diamanti-Kandarakis et al., 2009). Among the most concerning foodborne contaminants are the naturally occurring toxins, such as mycotoxins, and specifically aflatoxins (El Khoury et al., 2019). The current section focuses on two representative foodborne contaminants—the mycotoxin aflatoxin B1 (AFB1) and the herbicide ATZ—and discusses their toxic effects on bovine spermatozoa which carry over to the developed embryo.

## Effect of foodborne contaminants on male reproduction

Studies in occupationally exposed humans, as well as animals, have shown that pesticides may cause pathological changes in the male reproductive system, such as testicular damage, decreased spermatogenesis, and reduced semen quality (Zamkowska et al., 2018). Pesticides and certain toxins, including mycotoxins, may pass through the blood–testis barrier and negatively affect spermatogenesis (Ataman et al., 2014; Perry, 2008). Nevertheless, the direct effect of these foodborne contaminants on spermatozoa has been less studied, and the existing data are limited to basic parameters, such as spermatozoon concentration and motility. Moreover, the mechanism by which foodborne contaminants induce damage in spermatozoa is not fully understood.

**Figure 2:**
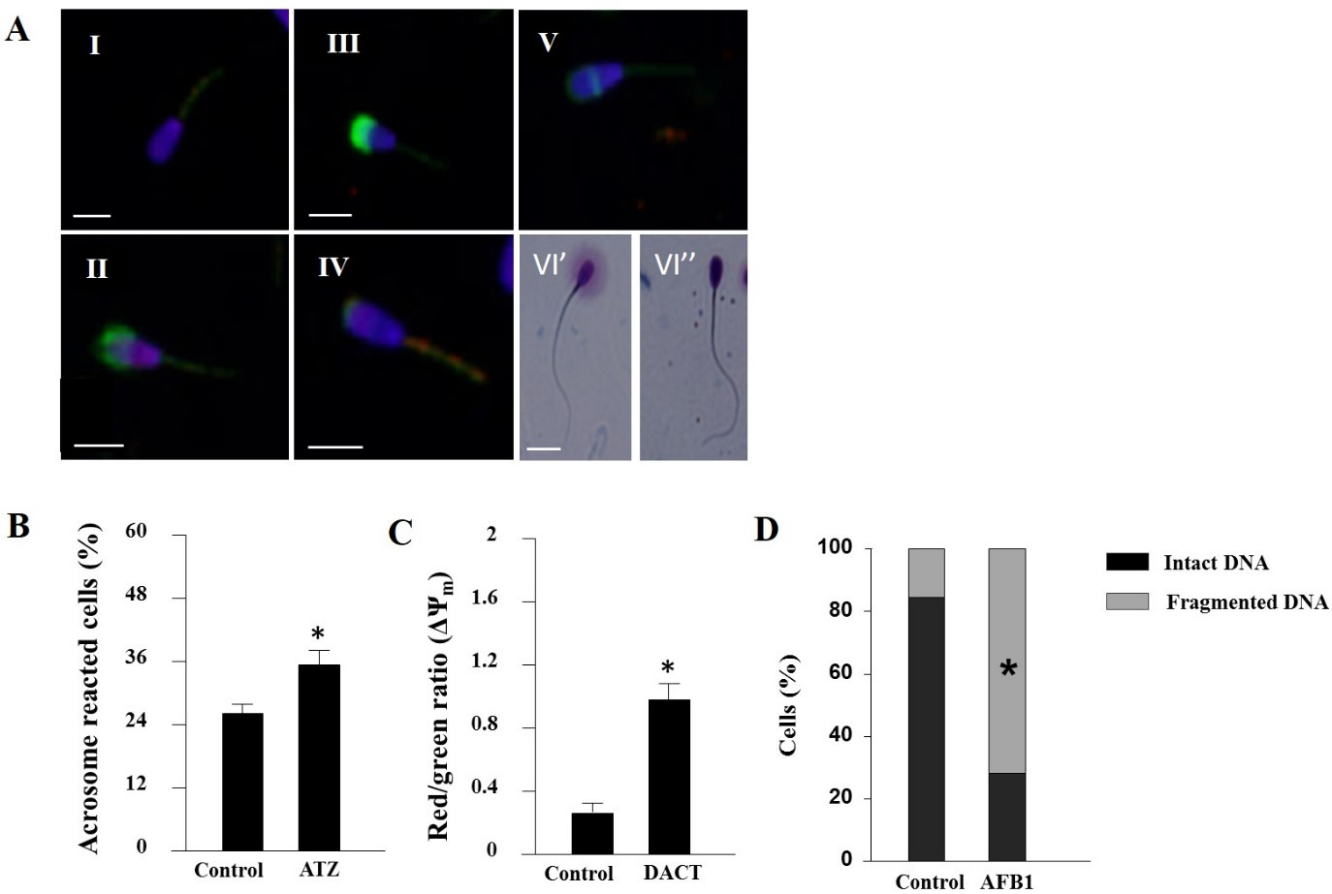
Effect of foodborne contaminants on spermatozoa. (A) Epifluorescence photomicrography of sperm cells stained with fluorescent probes: (I) live spermatozoon, nucleus stained with 4′,6-diamidino-2-phenylindole (DAPI; blue); (II) dead spermatozoon with damaged plasma membrane (propidium iodide staining, purple) and damaged acrosome (fluorescein-conjugated *Pisum sativum* agglutinin [FITC–PSA] staining, green); (III) live spermatozoon with damaged acrosome membrane (green); (IV) acrosome-reacted sperm cell with residual upper staining (green) and high mitochondrial membrane potential stained by JC-1 (red); (V) acrosome-reacted sperm cell with residual equatorial FITC–PSA staining (green); (VI) spermatozoon DNA fragmentation determined by Halosperm kit: (VI') spermatozoon with intact DNA expresses large halo and (VI”) spermatozoon with fragmented DNA expresses small or no halo. Scale bars = 10 µM (adopted with permission from Komsky-Elbaz and Roth, 2018). Spermatozoa were isolated from fresh ejaculate and incubated for 4 h without or with (B) 0.1 µM ATZ, presented is the proportion of acrosome-reacted cells  (FITC–PSA); (C) 1 µM DACT, mitochondrial membrane potential (ΔΨm) is presented as the mean proportion of red-stained (high potential) to green-stained (low potential) (JC-1) (adopted with permission from  Komsky-Elbaz and Roth, 2017); (D) 10 µM AFB1, spermatozoon DNA fragmentation determined by Halosperm kit (adopted with permission from Komsky-Elbaz and Roth, 2018). Data are presented as mean proportion ± SD, *P < 0.05.

Sperm fertilization potential depends on multiple factors. Among these are the integrity and functionality of sperm membranes, which indicate the sperm's viability and fertilization potential (Figure 2A; Komsky-Elbaz and Roth, 2018).

- *Plasma membrane* Damage to plasma membrane integrity may reduce spermatozoa's viability and thus reduce their fertilization capacity (Komsky-Elbaz and Roth, 2017). Various EDCs, such as BPA and phthalates, have been reported to decrease spermatozoon viability in humans (Pant et al., 2011; Knez et al., 2014), and the herbicide, fenoxaprop-ethyl, in porcine (Betancourt et al., 2006).
- *Acrosome integrity* Disruption of spermatozoon acrosomal membrane integrity leads to loss of its ability to undergo acrosome reaction (AR), resulting in reduced competence to attach to and penetrate the zona pellucida and fertilize the oocyte. Pseudo-AR (i.e., a non-controlled event which occurs via unknown factors such as environmental contaminants) can also lead to loss of fertilization ability (Wiser et al., 2014).
- *Mitochondrial function* Alterations in mitochondrial function are associated with physiological dysfunction, including male infertility (Ramalho-Santos et al., 2009). In humans, loss of ΔΨm is associated with low spermatozoon quality and caspase activation, which might eventually lead to apoptosis. Spermatozoa with altered ΔΨm generally have reduced fertilization potential and exhibit excess production of ROS (Espinoza et al., 2009). Various environmental compounds, such as EDCs, have been shown to induce cellular stress and lead to a transient increase in ΔΨm, i.e., hyperpolarization (Hüttemann et al., 2008), thereby leading to the generation of ROS (Saradha and Mathur, 2006). In turn, ROS can directly attack unsaturated fatty acids on the spermatozoon membrane, induce lipid peroxidation, damage membrane integrity, and eventually reduce the spermatozoa's fertilization potential (Lavranos et al., 2012). Mammalian spermatozoon membranes are rich in polyunsaturated fatty acids and thus are more susceptible to ROS attack, resulting in decreased sperm motility and viability, leading to infertility (Saradha and Mathur, 2006).
- *DNA fragmentation* The proportion of sperm with DNA fragmentation correlates with male fertility and is considered a practical parameter for characterizing semen quality (Sergerie et al., 2005). DNA molecules are a known target for various drugs, pesticides and toxic compounds (Ma et al., 2017; Virro et al., 2004). DNA damage was shown in subfertile males with increased BPA concentration in the urine (Meeker et al., 2010). In humans, increased DNA damage was documented after environmental and occupational exposure to the pesticide fenvalerate (Bian et al., 2004) and to a mixture of organophosphorous substances (Sánchez-Peña et al., 2004).
- *Epigenetic modifications* The environmental effect on the gametes is not limited to the obvious physiological and genetic effects. Epigenetic modifications and DNA methylation in the germline can be altered by various environmental factors (Gely-Pernot et al., 2015; Siddeek et al., 2018). Changes in differentially methylated regions were recorded in spermatozoa exposed to the fungicide vinclozolin (Skinner et al., 2019). Alterations in spermatozoa's DNA-methylation profile were reported upon paternal exposure to PBA and phthalates (Manikkam et al., 2013).

## Effect of aflatoxins on the spermatozoa

Aflatoxins are low-molecular-weight compounds produced mainly by the fungi Aspergillus flavus and Aspergillus parasiticus (Dai et al., 2017; Kew, 2013). Under humid conditions, these fungi grow on food grains, fruit and nuts, among others (Schenzel et al., 2012; Strosnider et al., 2006). Aflatoxin contamination can occur at any stage of the food chain (Verma, 2004), from preharvest to storage (Iimura et al., 2017; Shuaib et al., 2010). According to the US Food and Drug Administration the allowed amount of aflatoxins in human feed, is 4 to 30 ppb (0.01–0.1 μM), while grains for animal feeding can have up to 300 ppb (1 μM) (Williams et al., 2004).

Of the aflatoxins, AFB1 is considered the most toxic to mammals, owing to its hepatotoxic, teratogenic, mutagenic and immunosuppressive properties (Raisuddin et al., 1993; Shen et al., 1994; Meissonnier et al., 2008). AFB1 is also defined as an EDC as it affects the cytochrome P450 enzymes, which are involved in steroid synthesis (Storvik et al., 2011). Consumption of AFB1 leads to rapid metabolism into several metabolites, including aflatoxin M1 (AFM1).

Aflatoxins are of great concern to public health because they accumulate in the body and can be found in edible tissues, such as liver and muscles, as well as in animal food products such as milk and eggs (Fan et al., 2015; Giovati et al., 2015; Monson et al., 2016; Rajkovic et al., 2007; Verma and Nair, 2001; Yuan et al., 2016). Moreover, aflatoxins have been found in human maternal breast milk, and maternal and cord blood (Shuaib et al., 2010), and can apparently enter the developing fetus in humans and animals (Verma, 2004). AFM1 can also be found in milk and in other dairy products. It is resistant to heat and not fully inactivated by pasteurization or sterilization (Becker-Algeri et al., 2016). In the USA and some Asian countries, the maximum residual level of AFM1 in milk is 0.5 ppb (Becker-Algeri et al., 2016). The Israeli Public Health Services – Food Control Services (2008-2012) have reported milk samples containing AFM1 at levels of 0.08–0.10 ppb.

Although aflatoxins are considered one of the major risk factors for infertility (Gong et al., 2016), there is limited information on their effect on the male gametes (i.e., spermatozoa), in particular following exposure to allowable regulated levels. Evaluation of semen samples revealed the presence of aflatoxins in 8% of the samples collected from fertile men, compared to 40% of those collected from infertile men (Ibeh et al., 1994). Human exposure to aflatoxin is associated with abnormal spermatozoon count and morphology, as well as reduced motility (Ibeh et al., 1994; Nduka et al., 2001). Administration of 50 µg/kg BW AFB1 daily to mice for 45 days led to upregulation of genes involved in cell differentiation, immunity and the extracellular matrix in the testes. These alterations led to disruption of androgen production in Leydig cells (Austin et al., 2012). In rats, a dose-dependent decrease in the number of spermatozoa that developed in the seminiferous tubules was observed following administration with 0.8–3.2 ppm AFB1 for 48 days (Hasanzadeh and Rezazadeh, 2013). Rats administered 10–50 µg AFB1 for 60 days showed decreased steroidogenesis, and reduced reproductive organ weight, sperm quantity, and sperm quality (Supriya et al., 2014). Oral administration of AFB1 (50 µg/kg BW per day for 35 days) resulted in spermatotoxic effects on mouse epididymal sperm, expressed as reduced sperm concentration and motility and increased sperm abnormalities (Agnes and Akbarsha, 2003). Intramuscular injection of AFB1 (0.2–0.25 mL/day for 55 days) into male rats resulted in extrusion of the outer dense fibers and axonemal microtubule doublets of the sperm flagellum (Faisal et al., 2008a), suggesting negative effects on spermatozoa throughout the early stages of spermatogenesis.

In bovine, exposing spermatozoa to 10 µM AFB1 for 4 h decreased their viability, but did not activate the AR. In addition, hyperpolarization of the mitochondrial membrane was documented (Komsky-Elbaz et al., 2018). Direct interaction of AFB1 with the cells' DNA, generating reversible non-covalent interactions, was demonstrated (Ma et al., 2017). It was suggested that binding of AFB1 to the DNA of mammalian spermatogenic cells (Sotomayor and Sega, 2000) and epididymal spermatozoa (Faisal et al., 2008b) interferes with normal spermatogenesis, resulting in the production of abnormal spermatozoa and reduced fertilization. Exposure of bovine spermatozoa to 10 µM AFB1 significantly increased the proportion of DNA damage (Figure 2D; Komsky-Elbaz et al., 2018).

To date, very few studies have addressed the effect of mycotoxins on the male reproductive epigenome (El Khoury et al., 2019). In porcine, AFB1 at 50 µM induced alterations in histone, DNA methylation and apoptosis, and increased ROS levels (Liu et al., 2015). A recent proteomics approach to explore possible mechanisms by which AFB1 induces alterations in spermatozoa revealed differential expression of multiple proteins related to several cellular pathways and biological processes, and associated with genetic and epigenetic processes (Komsky-Elbaz and Roth, 2019).

## Effect of ATZ on the spermatozoa

ATZ is one of the best known chlorotriazine herbicides, extensively used to control growth of broadleaf and grassy weeds in agricultural crops (Severi-Aguiar and Silva-Zacarin, 2011). ATZ is considered a ubiquitous environmental contaminant, as it is frequently detected in ground and surface water, as a result of its mobility in the soil (Barr et al., 2007). ATZ has been banned for use in Europe since 2004, but is still utilized in about 70 countries, including US, Brazil, Argentina, Mexico, China, and Israel (LeBaron et al., 2008). Once it enters the body, ATZ is rapidly metabolized in the liver by cytochrome P450 enzymes into several metabolites, which are detected in the urine, serum and various tissues (Barr et al., 2007; Dooley et al., 2013; Ross et al., 2009); thus, an indirect effect of ATZ through its metabolites cannot be ruled out. In mammals, the major and most frequently detected metabolite is diaminochlorotriazine (DACT), shown to induce oxidative stress and disrupt endocrine function (Forgacs et al., 2013; Jin et al., 2014). DACT forms covalent adducts with various proteins, presumably as a chemical-induced toxicity step (Dooley et al., 2008).

Studies in humans have provided evidence for the transfer of trace amounts of ATZ to the circulation via the food chain (Diamanti-Kandarakis et al., 2009; Peighambarzadeh et al., 2011). For instance, ATZ has been detected in human amniotic fluid (0.6 µg/L) and urine (0.1 µg/L) (Bradman et al., 2003). Humans, wildlife, and domesticated animals can be exposed to ATZ via consumption of contaminated food or water, inhalation of pesticide spray, or absorption through the skin (Barr et al., 2007; Chevrier et al., 2011). Several studies have suggested that ATZ can alter reproductive functions, even at low, ecologically relevant doses (0.1–3 µg/L) (Abarikwu et al., 2010; Hayes, 2005; Jin et al., 2010). In amphibians, ATZ has a demasculinizing/feminizing effect (Hayes, 2005). Studies in rats have shown that ATZ elicits depletion of the antioxidant defense system in the testes, which in turn leads to oxidative stress (Abarikwu et al., 2010; Jin et al., 2010).

Gely-Pernot et al. (2015) reported that ATZ interferes with the process of meiosis in male mice. Interruption of meiosis, a key step in gametogenesis, may lead to the production of abnormal spermatozoa and reduced sperm quality, as found in ATZ-treated animals (Abarikwu et al., 2015; Feyzi-Dehkhargani et al., 2012). Supplementation of male goats with 15 mg/kg BW ATZ daily for 6 months impaired morphology, viability, ΔΨm, and cell lipid composition of ejaculated and epididymal spermatozoa (Komsky-Elbaz et al., 2019a). Daily administration of 25 mg/kg BW ATZ to mice impaired the production of spermatozoa by interfering with meiosis in the testes (Gely-Pernot et al., 2015). In rats, daily administration of 120 mg/kg BW ATZ depleted the antioxidant defense system (Abarikwu et al., 2010) and induced oxidative stress (Abdel Aziz et al., 2018) in the testes.

ATZ has been shown to decrease viability and increase the occurrence of pseudo-AR in bovine (Figure 2B; Komsky-Elbaz and Roth, 2017) and boar (Maravilla-Galván et al., 2009) spermatozoa. In this context, daily administration of 15 mg/kg BW ATZ to male goats for 6 months impaired the ejaculated spermatozoa’s membrane composition, expressed as an increased proportion of saturated fatty acids and reduced proportion of polyunsaturated fatty acids, which were associated with spermatozoon quality (Komsky-Elbaz et al., 2019a). Mitochondrial membrane hyperpolarization was documented in ejaculated bovine spermatozoa after 4 h exposure to ATZ or DACT (Figure 2C; Komsky-Elbaz and Roth, 2017). In *Drosophila* spermatozoa, ATZ has been shown to affect mitochondrial electron transport (Hase et al., 2008). In addition, ATZ is suggested to inhibit mitochondrial function in human spermatozoa by binding to F1F0–ATP synthase (Hase et al., 2008). In terms of practical aspects, i.e., artificial insemination, exposure to ATZ (0.1–1 µM) or its metabolite, DACT (1–10 µM), reduced spermatozoa's cryotolerance and survival post-thawing, by interfering with membrane integrity (Komsky-Elbaz et al., 2019b).

Epigenetic interference with spermatogenesis was documented in male mice (Gely-Pernot et al., 2015). Exposure of rat to 25 mg/kg BW/day ATZ was shown to promote epigenetic transgenerational inheritance, expressed by sperm differential DNA methylation regions over three generations (McBirney et al., 2017). Significant transgenerational differences in expression of genes involved in DNA methylation were reported in Medaka fish exposed to 5 or 50 μg/L ATZ (Cleary et al., 2019). Zebrafish larvae exposure to 3 ppb ATZ through embryogenesis lead to decreased expression of DNA methyltransferase and decreased DNA methylation levels (Wirbisky-Hershberger et al., 2017). Evaluation of the proteome of spermatozoa exposed to ATZ or DACT revealed differential expression of multiple proteins related to several cellular pathways and biological processes, associated with genetic and epigenetic processes (Komsky-Elbaz et al., unpublished data).

## Carryover effect of foodborne contaminants from the sperm to the embryo

During fertilization, the spermatozoon delivers paternal components to the oocyte which are crucial for oocyte activation, zygote formation, and further embryonic development (Dogan et al., 2015). Thus, paternal exposure to environmental contaminants plays a critical role in the offspring’s health (Siddeek et al., 2018). In-vitro fertilization of rat oocytes with epididymal sperm capacitated with AFB1 (2–16 ppb) resulted in significantly lower fertilization rates relative to fertilization with untreated sperm (Ibeh et al., 2000). In bovine, the proportion of oocytes that were fertilized and cleaved to 2- to 4-cell-stage embryos was lower after fertilization with spermatozoa exposed to 10 µM AFB1 compared to non-treated spermatozoa, but blastocyst-formation rate was relatively fair (Komsky-Elbaz et al., 2018). One possible explanation is that although the spermatozoa were exposed to AFB1 through capacitation, fertilization was performed by those that had not been impaired.

The spermatozoa's contribution to the embryo is not limited to the paternal DNA; it also includes epigenetic marks on the DNA, as well as RNA and proteins that are transferred through fertilization (Castillo et al., 2018). About 108 sperm-originated proteins have been identified in the human blastocyst (Castillo et al., 2018), and these might be crucial for embryonic development and health (Oliva et al., 2015; Rando, 2012). It is also possible that the reduced cleavage rate reported for AFB1-treated spermatozoa expresses a delay in embryonic development rather than fertilization failure. In support of this assumption, it has been recently reported that sperm with DNA damage can fertilize the oocyte (Bungum et al., 2004). Moreover, soon after fertilization, a repair process begins in the oocyte (Uppangala et al., 2016), suggesting that the embryo can repair DNA damage of sperm origin. On the other hand, it has been reported that a high proportion of sperm with DNA damage is associated with altered metabolism in the developing blastocyst (Uppangala et al., 2016). These findings suggest that damaged DNA not only reduces sperm fertilization competence, but also negatively affects embryonic development (Ioannou et al., 2016) and increases the number of embryonic cells undergoing apoptosis (Simões et al., 2013). Exposure of spermatozoa of the marine invertebrate *Galeolaria caespitosa* to dibutyl phthalate inhibited fertilization and embryogenesis (Lu et al., 2017). Although sperm with damaged DNA can fertilize the oocyte, genomic instability in the developed embryo cannot be ruled out (Adiga et al., 2010). Evaluating the transcriptome profiles of blastocysts derived following in-vitro fertilization with AFB1-treated spermatozoa revealed a significantly different profile from that of the control, suggesting a carryover effect of AFB1 from the spermatozoa to the developing embryo (Komsky-Elbaz and Roth, unpublished data).

Traditional studies claim that inheritance from one generation to the next occurs solely through genetic information. However, there is increasing evidence of an important role for epigenetic factors in the transmission of certain phenotypes from parents to their offspring. Environmentally induced epigenetic alterations can be inherited through the male gametes (Wei et al., 2015) and might regulate gene expression at transcriptional as well as post-transcriptional levels in the early developing embryo (Castillo et al., 2018). For instance, paternal exposure to EDCs may cause alterations in the spermatozoa's RNA, and some of these changes might persist in the offspring (Uppangala et al., 2016). In mice, paternal prediabetes altered overall methylome patterns in spermatozoa, with a large portion of differentially methylated genes overlapping with genes of pancreatic islets in the offspring (Wei et al., 2014). Thus, it is highly possible that alterations in the transcriptomic profile of embryos derived after fertilization with AFB1-treated spermatozoa will include not only genetic but also epigenetic alterations. This point should be further evaluated.

## Summary

There is a wide range of evidence for the potential hazard associated with exposure of spermatozoa to foodborne contaminants. The damage is already initiated in the testes during spermatogenesis, persists during the sperm's passage through the epididymis and finally, is expressed in the mature spermatozoa. Exposure of the spermatozoa to contaminants may also occur after ejaculation, in the female reproductive tract. The damage seems to be multifactorial in nature, involving alterations in the spermatozoa’s membranes, DNA and proteome. Interestingly, different contaminants do not necessarily have the same effect on spermatozoa, suggesting different modes of action. Evidence of the damage in the spermatozoa carrying over to the developing embryo is of great concern, and warrants further investigation.

## General Summary

**Figure 3:**
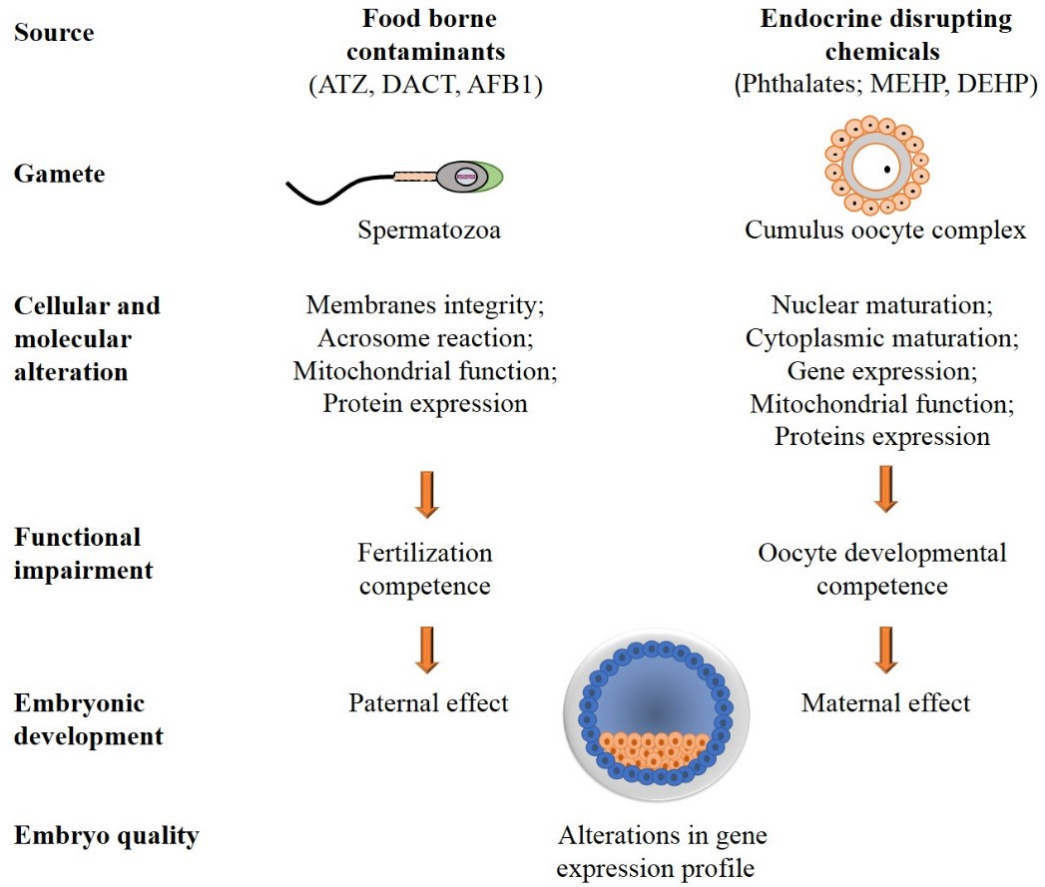
Schematic illustration of the effects of EDCs and foodborne contaminants on female and male gametes. ATZ and AFB1 induce alterations in the spermatozoa. These include cellular and molecular alterations, which in turn impair fertilization competence. MEHP induces cellular and molecular alterations that affect oocyte developmental competence. Alterations in both male and female gametes are further expressed in the developed blastocyst.

This review provides a broad screening of the information on the effects of EDCs and foodborne contaminants on both the oocyte and spermatozoa (Figure 3). When the oocyte is exposed to MEHP, deleterious effects are further recorded in the developed embryos. Similarly, exposing spermatozoa to foodborne contaminants further affects the formed embryos. While this information is directly related to the presented environmental contaminants, these data might also be relevant to other EDCs. Exploring the risk associated with exposing animals to environmental contaminants might lead to better management for reproductive health preservation.
